# Strychnia in Whisky

**Published:** 1874-08

**Authors:** 


Strychnia in Whisky.—The editor of the *Druggists' Circular* does not believe the popular temperance myth of the presence of strychnine in whisky. He says:

"If strychnia has the power of increasing the yield of whisky from a given amount of 'mash,' it is a new property of that alkaloid, which is not mentioned in any work on chemistry. Even admitting that strychnia could help the operation in any way, and increase the production, the whole of the poison would remain in the still, for strychnia is not volatile at the temperature of the distillation of whisky. The whole thing is an absurd story, invented, no doubt, to frighten people. Why not say the truth at once? Whisky kills more than enough people when pure; it needs no assistance from other deadly poisons.

